# Mobile Computing Technologies for Health and Mobility Assessment: Research Design and Results of the Timed Up and Go Test in Older Adults

**DOI:** 10.3390/s20123481

**Published:** 2020-06-19

**Authors:** Vasco Ponciano, Ivan Miguel Pires, Fernando Reinaldo Ribeiro, María Vanessa Villasana, Rute Crisóstomo, Maria Canavarro Teixeira, Eftim Zdravevski

**Affiliations:** 1 R&D Unit in Digital Services, Applications and Content, Polytechnic Institute of Castelo Branco, 6000-767 Castelo Branco, Portugal; vasco.ponciano@ipcbcampus.pt (V.P.); fribeiro@ipcb.pt (F.R.R.); 2 Altranportugal, 1990-096 Lisbon, Portugal; 3 Instituto de Telecomunicações, Universidade da Beira Interior, 6200-001 Covilhã, Portugal; 4 Department of Computer Science, Polytechnic Institute of Viseu, 3504-510 Viseu, Portugal; 5 Faculty of Health Sciences, Universidade da Beira Interior, 6200-506 Covilhã, Portugal; maria.vanessa.villasana.abreu@ubi.pt; 6 Polytechnic Institute of Castelo Branco, 6000-084 Castelo Branco, Portugal; crisostomo.rute@ipcb.pt; 7 UTC de Recursos Naturais e Desenvolvimento Sustentável, Polytechnique Institute of Castelo Branco, 6001-909 Castelo Branco, Portugal; ccanavarro@ipcb.pt; 8 CERNAS—Research Centre for Natural Resources, Environment and Society, Polytechnique Institute of Castelo Branco, 6001-909 Castelo Branco, Portugal; 9 Faculty of Computer Science and Engineering, University Ss Cyril and Methodius, 1000 Skopje, North Macedonia; eftim.zdravevski@finki.ukim.mk

**Keywords:** Timed-Up and Go test, sensors, mobile devices, accelerometer, magnetometer, pressure sensor, feature detection, diseases, older adults

## Abstract

Due to the increasing age of the European population, there is a growing interest in performing research that will aid in the timely and unobtrusive detection of emerging diseases. For such tasks, mobile devices have several sensors, facilitating the acquisition of diverse data. This study focuses on the analysis of the data collected from the mobile devices sensors and a pressure sensor connected to a Bitalino device for the measurement of the Timed-Up and Go test. The data acquisition was performed within different environments from multiple individuals with distinct types of diseases. Then this data was analyzed to estimate the various parameters of the Timed-Up and Go test. Firstly, the pressure sensor is used to extract the reaction and total test time. Secondly, the magnetometer sensors are used to identify the total test time and different parameters related to turning around. Finally, the accelerometer sensor is used to extract the reaction time, total test time, duration of turning around, going time, return time, and many other derived metrics. Our experiments showed that these parameters could be automatically and reliably detected with a mobile device. Moreover, we identified that the time to perform the Timed-Up and Go test increases with age and the presence of diseases related to locomotion.

## 1. Introduction

### 1.1. Background

The increasing age of the world population has promoted research in several areas and advances in different types of sensors, which have contributed to the evolution of healthcare assessment methodologies [1]. The increased life expectancy has led to growing interest and the need for solutions that can improve the quality of life of the elderly. In Europe, the aging rate was 125.8% in 2017, and 94.1% in 2001 [2,3,4,5].

Mobile computing technologies made it possible to aid individuals with different health statuses. They now include multiple sensors, which can be used for a verity of diverse functions [6]. The magnetometer and the accelerometer are essential because they facilitate the acquisition of physical and biological data from the user [7,8,9]. Moreover, these sensors can support the analysis of bodily functions like gait [10,11]. Furthermore, combining mobile computing technologies with external sensors can promote older people’s quality of life [12]. However, in such studies, there are challenges related to choosing adequate tests, and interpretation and analysis of the collected data [13,14,15,16,17].

Embedded sensors may help to monitor the different functional tests with the detection of different types of movements [18,19,20,21,22]. The Timed-Up-and-Go test is a quick and straightforward clinical test for assessing lower extremity performance related to balance, mobility and fall risk in the elderly population and people with pathologies (i.e., Parkinson’s disease, amyotrophic lateral sclerosis, in post-stroke patients, in patients with orthopedic pathologies, and cardiovascular incidents) [23,24,25,26,27,28]. Aging effects can be identified with the Timed-Up-and-Go test, and it could be supplemented with smart technology to be used in clinical practice [29]. The automation of the measurement of sensor data when performing the Timed-Up and Go test can be valuable, particularly in older adults [30,31]. Some approaches, such as [32], make it possible to perform the Timed-Up and Go test using low-cost devices in a real-time setting with reduced needs of processing capabilities to be used in commonly used devices.

### 1.2. Motivation

The Timed-Up and Go test can provide a practical analysis of the degree of prevalence and level of certain diseases [33]. With this test, clinicians can assess physical conditions by evaluating the way the individual walks, and the time it takes to perform the analysis. Therefore, this test allows the medical team to assess whether the individual has an accelerated degree of disease development or is in the initial state [34].

Furthermore, the Timed-Up and Go test can be used in individuals with neurological diseases [35]. This test allows for the evaluation of their reaction time. It is possible to assess whether they get up quickly or still stop for a long time. Moreover, it is possible to evaluate whether the individual walks in a straight line or cannot maintain the correct direction [36,37]. Therefore, this test can also provide a practical assessment of cognitive problems that do not allow him to follow the right path.

This test is widely used in assessing a patient’s recovery process associated with diseases that have affected their mobility [38]. The data collected in this test support the evaluation of patient recovery to establish standards related to the reaction time, test time, angular derivation, and walking strength that an individual with different degrees of the disease might have [39].

This paper’s motivation is to present a cost-effective method for the automatic measurement of the Timed-Up and Go test using sensors available on common smartphones. This document also states the calculation of numerous features that aim to create a reliable dataset for pattern recognition on specific health symptoms. Moreover, this study provides a comparative analysis of different subjects, which live in nursing homes separated by age, institution, and various diseases of people, finalizing with the comparison with the other results available in the literature to state the useful contribution of the proposed approach.

Finally, the major challenge with this is related to the definition of the best positioning of the sensors for the correct data acquisition. Thus, it affects the measurement of the different results of the Timed-Up and Go test, e.g., in case the experiments are performed under adverse conditions, the probability of having the incorrect measurement of the results is very high. Technological constraints may also affect the data acquisition and processing, such as low memory, power processing, connectivity, network, and battery constraints of the mobile devices [40,41]. Previously, we explored and presented the positioning of the sensors available in a mobile device or connected in a Bitalino device with the preliminary results in [42,43].

### 1.3. Prior Work

There are some studies available in the literature that involved the calculation of the different features related to the Timed-Up and Go test for further conclusions about the performance of the test. The inertial sensors, e.g., accelerometer, magnetometer, and gyroscope, available in a mobile device may be used to evaluate the benefits of the training based on the Timed-Up and Go test, calculating the velocity and the time of a sit-to-stand transition [44].

Fall risk assessment based on wearable inertial sensors was performed based on an instrumented Timed-Up and Go test in [45], relying on a variety of features, as summarized in Table A1. The types of gait and balance were evaluated with a similar set of features in [46]. The accelerometer sensor was used for the identification and measurement of the duration of each stage of the Timed-Up and Go test in individuals with spinal cord injury [47]. The different phases were also evaluated in [48] with an accelerometer sensor, measuring the mobility angles, and the average of the sit-to-stand transition time in frail elderly individuals with Parkinson’s disease. In [49], the measurement of the Timed-Up and Go test results was performed with an accelerometer sensor for fall risk assessment. The different phases of the test for people with Parkinson’s disease were analyzed in [50] and [51]. In [52], patients with Parkinson’s disease were analyzed during a walking activity to measure the duration of the test. A smartphone application suite for assessing mobility is presented in [53]. Whether the individual was sitting during the Timed-Up and Go test is investigated in [32]. The authors of [54] perform analysis, mainly focusing on people with frailty syndrome. A wearable system for assessing mobility in older adults is presented in [55], relying on a variety of statistical features. Similarly, a wearable system for measuring the probability of human falls is introduced in [56], while [17] is concerned with identifying the reasons for falls. In [57], the authors show that the mobile device accelerometer can study and analyze the Romberg test’s kinematic between frail and non-frail older adults.

In summary, Parkinson’s disease was analyzed in six studies [46,48,50,51,52,58], Arthrosis [45,53] and Frailty syndrome [54,57] in two studies, and Dizziness [45], hypertension [45], polypharmacy [45], and spinal cord injury [47] in one study each.

### 1.4. Structure of the Study

The remainder of this paper is organized as follows: Section 2 presents the methods used for the development of the proposed analysis, including the study design and participants, description of the Timed-Up and Go test, the data acquisition and processing methods used, and the statistical analysis performed in this study. The mobile application developed for data acquisition, the requirements, and the statistical analysis are presented in Section 3. Furthermore, Section 4 offers a discussion on the main findings, limitations, and comparison with our study’s prior work. In the end, Section 5 presents the conclusions of this study.

## 2. Methods

### 2.1. Study Design and Participants

We selected Android as the operating system for data collection software development as it is open-source software and a market leader. Moreover, we chose the external Bitalino sensors for their appropriate use in research projects in this research domain [59]. This technology could facilitate the creation of significant datasets for health assessment that can be used to support decision-making in medical diagnostics. The mobile device was incorporated in a sports belt to be worn on the waistline. The start of the Timed-Up and Go test was indicated by a sound alarm using the mobile application. The chair incorporated a pressure sensor to register the moment when the older adult re-acted to this sound. The volunteer had to walk for 3 m, go back, and sit down again. All the data were collected on the mobile device, and, after test finalization, a text file was sent to the Cloud by using the FireBase service. Different mobile devices were used for data acquisition to compare the different frequencies of the data acquisition, which verified that the XIAOMI MI 6 was one of the devices that more accurately acquired the different types of data. As the experiments were controlled, we used the same device for final data acquisition and analysis. The data acquisition showed an influence of the environment and varied with the place for data acquisition. It was associated with the study of older adults with different health conditions and ages and resulted in the creation of a dataset with diverse and heterogeneous data.

The data acquired were processed with the Java programming language to extract the different features for the statistical analysis. Firstly, the pressure sensor is used to measure the reaction and total test time. Secondly, the magnetometer sensors are used to extract the total test time, turning around instant by the magnitude of the vector and turning around instant by the absolute value of the z-axis. Finally, the accelerometer sensor is used to extract the reaction time, total test time, duration of turning around, going time, return time, and the averages of the acceleration, velocity, force, and power during going and returning time. 

The proposed method was tested on 40 older adults with an age of 60- to 97-years-old (83.8 ± 7.95), privileging gender equality from four institutions, such as Centro Comunitário das Lameiras, Lar Aldeia de Joanes, Lar Minas, Lar da Misericórdia, and others. The “others” corresponds to an open group from different locations. They have several types of health complications, such as Parkinson’s disease, scoliosis, mobility, and cardiovascular problems, and dementia complications (presented in Table A2). The volunteers were institutionalized in nursing homes in the center of Portugal. The selection process was conducted in close collaboration with the nursing team. However, the inclusion criteria relied on mobility capabilities to perform the test. The individuals are randomly selected, and there is no relationship between the individuals and the team of this study. The volunteers were informed about all the specifications and goals of the experiments.

Furthermore, they signed an ethical agreement allowing us to share the results of the tests in an anonymous form. The agreement also provided the participants’ informed consent considering the risks and the objective of the study. Ethics Committee from Escola Superior de Saúde Dr. Lopes Dias at Polytechnic Institute of Castelo Branco approved the study with the number 114/CE-ESALD/2019.

Moreover, other information such as age and weight were provided to support the conclusions of the study. These data were guaranteed to be used in an anonymous form. The data were then measured using a feature extraction method that will be explained in Section 2.2. 

Only consistent data were considered in these results. The experiments were held between October and December 2019, and each volunteer underwent the test at three different times. These tests were conducted in an isolated environment to avoid any distractions, which could impact the results. Each institute provided the chair used in the experiments. The volunteers had different health states, some of them still healthy, had diseases related to the spine, such as multiple sclerosis, diseases related to the heart, arrhythmia, or angina pectoris, or illnesses associated with the mental health, such as Parkinson’s. These people had various health statuses and distinct degrees of progress for each disease, which indicated that the population’s health status was variable. Thus, the data collected were heterogeneous.

The mobile application acquired the data from the sensors at intervals of milliseconds, but it was converted to seconds to improve its readability. The collection process started with an audible signal. This sound signal represented the beginning of the data capture, which was recorded in text files and sent over the Internet using the Firebase service. Initially, the data were saved in text files. The accelerometer and magnetometer were tri-axis sensors, represented in four columns in the different files, including timestamps and one column for each axis of the sensors (x, y, and z). Further, the pressure sensor acquired the force performed with the user sitting on the chair. These sensors were complementary for the measurement of the different parameters of the Timed-Up and Go test.

### 2.2. Description of the Timed-Up and Go Test and Data Acquisition and Processing

The Timed-Up and Go test was developed in 1991 to examine functional mobility in the elderly [60,61]. This test allows the recognition of other different diseases, mainly related to walking activities. It has certain phases where it is possible to obtain different readings and calculations of various features, such as sitting on the chair, lifting from the chair, walking for three meters, reversing the march, walking another three meters toward the chair, and sitting on the chair. 

The data acquisition was performed with a mobile device equipped with accelerometer and magnetometer sensors, placed in a belt at the waist of the person, and two Bitalino devices, i.e., one with a pressure sensor placed on the back of the chair, and the other with one ECG and one EEG sensor placed in a belt at the chest of the individual.

Currently, only the data acquired from the pressure sensor and the sensors available in the mobile device are processed. Thus, different calculations are performed, including reaction time, time of the end of data acquisition, the total time of the test, turning instant, turning time, walking time, returning time, the average of the acceleration, speed, force, and power. The measurements of the speed, strength, and power are essential to detect some abnormalities in the actions of older adults.

### 2.3. Statistical Analysis

After the acquisition of the data from the sensors available in off-the-shelf mobile devices and the sensors connected to the Bitalino device, the data analysis was performed. Firstly, the data acquired by the pressure sensor were processed, extracting the reaction time and the total test time. Secondly, the data obtained by the magnetometer sensor were processed, extracting the start time, the end time, the instant and acceleration value of turning around by the Euclidean norm, and the instant and acceleration value of turning around by the minimum absolute value of the acceleration. Thirdly, the data acquired by the accelerometer sensor were processed, extracting the start, reaction, end, and total test times, the instant and duration of turning around, time of walking the first three meters, time to walk back to the chair, and the mean of the acceleration, velocity, force, and power during the walk for the first three meters and during the walk back to the chair.

After measuring the different variables, a statistical comparison between them was performed, analyzing and comparing the results to the averages of each institution, person, and healthcare disease. Also, descriptive statistics, normality tests, and the detection of outliers were performed. After checking the conditions and making sure we can apply ANOVA, we used it to compare averages between institutions and age groups. Thirdly, the results were analyzed by each disease. The ANOVA test was used for the dependence between the different variables to test the relation between the results obtained and the sample characteristics. ANOVA is a statistical test that allows the discovery of potential differences or relations between different variables useful in testing with the distinct features of human beings [62,63]. It will enable the assessment of possible ties and dependencies between different variables. As the Timed-Up and Go test is a physical test related to people’s physical conditions, different variables may be affected. 

## 3. Results

### 3.1. Data Acquisition with a Mobile Application

The mobile application was developed for Android devices using the Android Studio Integrated Development Environment (IDE). The mobile application has two main functionalities. On the one hand, this mobile application performs a continuous data collection using the built-in magnetometer and accelerometer sensors. The data are collected with a sampling rate of 1 kHz and 16 bits of precision. On the other hand, the mobile application handles the communication technologies required to receive data through Bluetooth from the Bitalino device with a pressure sensor but is also responsible for sending the collected data to the Firebase service for storage. The analysis showed that the mobile devices with embedded sensors provide reliability and automation in the Timed-Up and Go test, unlike traditional measurement methods that require manual measuring. 

### 3.2. Requirements

There are two different types of requirements verified for the performance of the experiments, i.e., one related to the environment and the other to the individual. For the execution of the Timed-Up and Go test, the individual should have the possibility to walk, stand-up, and sit-down on the chair independently. It needs a chair, a tape-measure for the identification of the place related to the three meters to walk, and an adhesive tape to mark the site where the individual should reverse the gait. Also, electrodes to position the EEG and ECG sensors in the individual, an adhesive tape to fix the pressure sensor on the chair, and two sports belts to carry the mobile device and the Bitalino device are used. 

### 3.3. Comparison of Different Acquired Data

There are a few options to measure the turning around instant, which are:

- The minimum value or amount of the magnitude of the vector of the accelerometer, calculated after the reaction time;
- The minimum absolute value of the z-axis of the magnetometer, calculated after the reaction time.

Based on the presented steps for the calculation of the turning around instant, the first moment of mobility, and the start time of the test can be measured by the accelerometer and the pressure sensor. 

Incidentally, the analysis performed in this paper includes several values. These are:

- Pressure sensor: reaction time, whole test time;
- Magnetometer: total acquisition time, turning around instant by the magnitude of the vector, turning around moment by the absolute value of *z*-axis;
- Accelerometer: reaction time, total test time, duration of turning around, going time, return time, the average acceleration during going time, the average acceleration during return time, the average velocity during going time, the average speed during return time, the average force during going time, the average force during return time, the average power during going time, the average power during return time;

Next, the presentation of these results by age (Section 3.3.1), by institution (Section 3.3.2), and by disease (Section 3.3.3) will be performed.

#### 3.3.1. Results by Age

**Figure 1:**
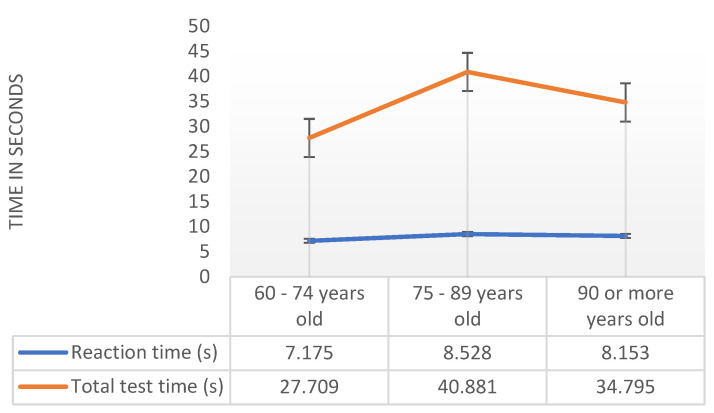
Analysis of reaction time and total test time with pressure sensor by age range.

After checking the requirements, we used the ANOVA test. We found out that there is no statistically significant difference (alpha = 0.05) between the three age groups for all variables/measurements of interest. Figure 1 shows the mean values for the different age ranges for the reaction time and total test time variables obtained with the pressure sensor. Thus, the results of the F-test, through the respective limited probability associated with the test statistic allowed us to conclude that the average values between the three age groups are statistically equal for the analysis for the magnetometer sensor, such as Pr (F > F-test) = 0.231 > 0.05 for the total test time variable, and Pr (F > F-test) = 0.815 > 0.05 for the reaction time variable. Therefore, we accept the null hypothesis that the averages are statistically equal. Although the averages are statistically equal, it is interesting to note that both for the reaction time and for the total variable test time, it is the younger individuals who have shorter times, as expected. However, the group of individuals in this age group is only five people, and the group of older individuals is only eight people. For statistically more relevant results, the population needs to be increased in future experiments.

**Figure 2:**
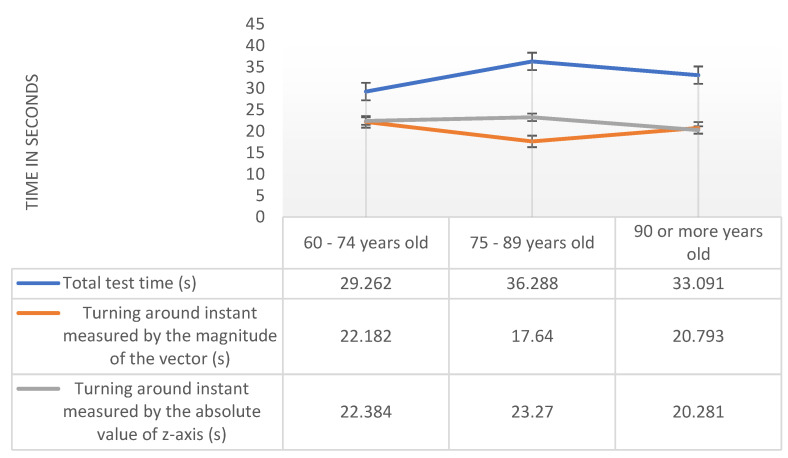
Analysis of total test time, turning around instant measured by the magnitude of the vector and turning around instant measured by the absolute value of the z-axis with the magnetometer sensor by age range.

Then, in Figure 2, we can observe the mean values for the different age range for total test time, turning around instant measured by the magnitude of the vector, and turning around moment measured by the absolute value of z-axis variables obtained with the magnetometer sensor.

The results of the ANOVA test, through the respective limit probability associated with the test statistic, allowed us to conclude that the average values between the three age groups are statistically equal for any of the variables under analysis for the magnetometer sensor, namely 32.88 (s) for total test time (Pr (F > F-test) = 0.637 > 0.05), 20.21 (s) for turning around instant measured by the magnitude of the vector Pr (F > F-test) = 0.772 > 0.05, and 20.28 (s) for turning around moment measured by the absolute value of z-axis variables obtained with the magnetometer sensor Pr (F > F-test) = 0.735 > 0.05.

#### 3.3.2. Results by Institution

Aiming to investigate any differences between the participating institutions in this study, we performed a set of ANOVA tests where alpha = 0.5. In cases when there is a statistically significant difference (*p* < alpha), we applied Tukey’s multiple comparison tests to identify homogeneous institutions. For conciseness, we only list the parameters which are statistically significantly different between the institutions (*p* < alpha).

Namely, the variables with a significant difference in the mean for different institutions are: total test time (s), the conclusion is that there are significant differences between institutions (*p*-value = 0.03 < alpha = 0.05). The total test time (s) by the pressure sensor, the turning around instant by the absolute value of z-axis (s) by the magnetometer, the total and return test times (s), the averages of velocity during going and returning time (m/s), and the averages of power during going and returning time (J), the total test, going and returning times (s), the average of velocity during return time (m/s), the total test and return times (s), and the averages of velocity and power during going time (m/s) by accelerometer and magnetometer.

Also, we concluded that the average values of all institutions are statistically equal for the reaction time, duration of turning around, the averages of acceleration, velocity, force, and power during going and returning times. The results of this analysis can show that more generic features are statistically equal in different institutions, and therefore might be useful for drawing general conclusions that apply to older adults in general.

#### 3.3.3. Results by Disease

At this stage, approximately 40 different pathologies associated with the subjects were identified. Some individuals have only one pathology, but others have more diseases and from very diverse areas, as shown in Table 1. Of the 40 individuals involved in the study, there are 11 patients with one pathology, nine patients with two pathologies, five patients with three pathologies, five patients with four pathologies, two patients with five pathologies, and only one patient with 6, 7, and 9 pathologies. We can also see the number of individuals identified by pathology and the classification of the respective pathologies by respective categories. This analysis reflects the great diversity of pathologies vs. individuals under study, which may make it difficult and even compromise inferential statistical analysis.

Also, it was not possible to read all sensors in the same way for all individuals, resulting in different numbers of samples for the different variables under study. As presented in Table 2, two groups were formed with the pathologies under analysis, including one for diseases directly related to mobility, and others with the other conditions found in the population.

**Figure 3:**
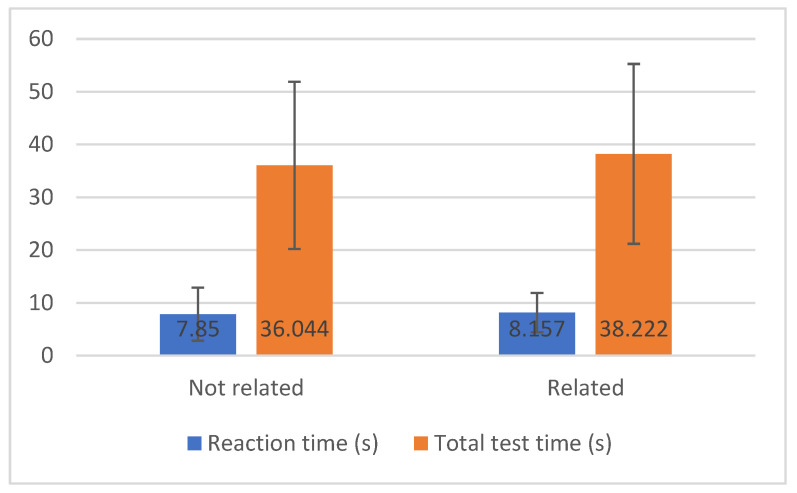
Analysis of reaction time and total test time with a pressure sensor.

In Figure 3, we can observe the mean and the standard deviation values for reaction time and total test time measured by the pressure sensor by groups of diseases related to mobility and not directly related to movement. Through using the Student’s *t*-test to compare two groups of independent samples, it was possible to assess whether there are statistical differences in the level of measurements made between individuals with diseases related to mobility and not associated with movement. 

First, we concluded the variances are homogeneous (Pr (F > F-test) = 0.079 > 0.05). With the Student’s *t*-test, it was possible to conclude that the reaction time (s) between the two groups of diseases not related and related to mobility is equal (Pr (|T| > *t*-test) = 0.838 > 0.05), and the average is statistically similar to 37.133 (s). Hence, it can be said that the 13 individuals with pathologies not related to mobility take less time to perform the test (36.044 vs. 38.222), but this difference is not statistically significant.

Furthermore, the same conclusions can be achieved from the total test time (s) that has identical variances between the groups of diseases not related and related to mobility ((Pr (F > F-test) = 0.960 > 0.05)), and the average is statistically equal (Pr (|T| > *t*-test) = 0.710 > 0.05).

**Figure 4:**
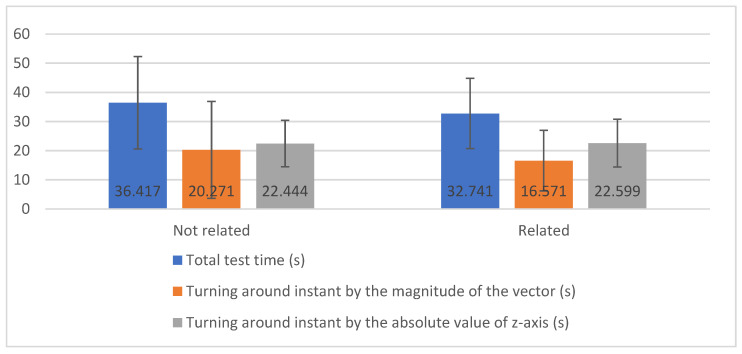
Analysis of total test time turning around instant by the magnitude of the vector and turning around instant by the absolute value of the z-axis with the magnetometer sensor.

In Figure 4, it is possible to observe the mean values for the total test time (s), turning around instant by the magnitude of the vector (s) and turning around instant by the absolute value of the *z*-axis (s) by magnetometer sensor by diseases related or not related to mobility.

With the application of the Student’s *t*-test for comparing the variables measured in the magnetometer sensor, by diseases related or not related to mobility, it was concluded that there are no significant differences in measurements between diseases related to mobility and not related to mobility. However, we can verify the following conclusions:

- The total test time (s) has homogeneous variances between the groups of diseases not related and related to mobility (Pr (F > F-test) = 0.459 > 0.05), and the average is statistically equal (Pr (|T| > *t*-test = 0.490 > 0.05);
- The turning around instant by the magnitude of the vector (s) has non-homogeneous variances between the groups of diseases not related and related to mobility (Pr (F > F-test) = 0.029 < 0.05), but the average is statistically equal (Pr (|T| > *t*-test = 0.642 > 0.05);
- The turning around instant by the absolute value of the z-axis (s) has homogeneous variances between the groups of diseases not related and related to mobility (Pr (F > F-test = 0.628 > 0.05), and the average is statistically equal (Pr (|T| > *t*-test = 0.961 > 0.05).

## 4. Discussion

### 4.1. Main Findings

The Timed-Up and Go test performed by the elderly population showed a considerable diversity of data because the participants had different types of diseases. The various physical states of each participant in the study demonstrated that the evaluation of the test was reliable with the use of sensors. Thus, the sensors available in the off-the-shelf mobile devices allowed practical data acquisition and further conclusions in real-time. Further, we used a pressure sensor for the reliable detection of the mobility of getting up from the chair. Thus, for additional findings, we extracted several features from the accelerometer and the magnetometer available in off-the-shelf mobile devices, and pressure sensors connected to the Bitalino device.

We anonymously collected the age and different diseases of people to consider during the test’s application in older adults. The data were analyzed from different viewpoints, including the measurements by each person, institution, and disease. It was proven that environmental conditions were essential for the reliability of the analysis of the results.

The conditions of the performance of the test, data acquisition, and network connection were adverse in two institutions, namely Lar Aldeia de Joanes and Lar Minas, as presented in Table 3. Considering the measurements performed by the data acquired from the magnetometer sensor, only the data obtained for 32 persons were reliable for further analyses. The relevant report was presented in Table 3. Thus, it is verified that the time measured by the magnetometer sensors was lower than the time measured with the data acquired from the pressure sensor. Considering the measurements performed using the data received from the accelerometer sensor, we concluded that the use of only the accelerometer sensor invalidated some tests in the calculation of the turning around instant. Only 16 persons performed the experiments with reliability, Table 3 presents the data. However, fusing these data with the measurements performed by the magnetometer sensor and using the turning around moment measured by the magnitude of the vector, we found that 22 persons performed the experiments with reliability. By using the turning around instant measured by the absolute value of the *z*-axis, we found that 33 persons performed the examinations successfully. Considering the measurements performed using the data acquired from the accelerometer sensor, we found that the use of only the accelerometer sensor invalidated some tests in terms of the calculation of the turning around instant. Thus, only three institutions performed the experiments with reliability, and only people with nine diseases were analyzed. However, fusing these data with the measurements performed by the magnetometer sensor, we concluded that the six institutions performed the experiments with reliability. Therefore, we find that the return time was higher than the going time with higher acceleration, velocity, force, and power during the return time. Thus, we concluded that the return time was higher than the going time with higher acceleration, velocity, force, and power during the return time. With the fusing of these data with the measurements performed by the magnetometer sensor and using the turning around moment measured by the magnitude of the vector, we analyzed 16 diseases. Using the turning-around instant measured using the absolute value of the *z*-axis, we analyzed 27 illnesses.

Some individuals reported an inconsistency between the different diseases and the results obtained by the values acquired using the various sensors, and this inconsistency could be attributed to the adverse conditions of the data acquisition. In general, older adults have more than one disease. Still, the best results obtained with the magnetometer were obtained in people with arthrosis disease, where the person only has arthrosis, and the other people have several diseases. The same problem was observed in the case of people with osteoarticular pathology, and prosthesis in the right humeral, where the going time was lower than that for the other people. In conclusion, the sensors might report bad data, and the findings might be argued. The other problem was that people with osteoarticular pathology and prostheses in the right humeral reported better results in the measurement of turning around than people with lumbar hernias and gastric ulcers. They were attributed to the fact that people with gastric ulcers had more than one disease, and people with several diseases reported higher times than the others.

To ensure that these data collection methodologies can be used to assess physical and functional performance in the clinic, this data should be valid, reliable, and with proper responsiveness, as has been demonstrated by the Timed-Up and Go test in a variety of conditions [64,65].

### 4.2. Limitations

As presented in Table 4, there are three possible origins of limitations found, such as individuals, environment, and technical. The older adults and environments for the different tests are heterogeneous. However, other technical barriers related to the Internet and Bluetooth connection availability, and synchronization between the various devices were found. The individuals performed the examination three consecutive times to avoid some problems, and the acquisition started at the same time in all devices.

### 4.3. Comparison with Prior Work

Different studies analyzed the performance of the Timed-Up and Go test with sensors to measure the various parameters. Still, only two studies [45,50] show the values of the measured parameters. These studies are not comparable with the values obtained in our study, because they only calculate the power. There are multiple literature surveys of the Timed-Up and Go test [60,64,66], but they do not explicitly consider the inclusion of older adults. It is also evident because of the discrepancy in the reported values of high power, which is uncommon for older adults who usually have low energy. As the people of other studies are younger, the power/energy used to perform the Timed-Up and Go test is higher than in our research, reporting −28,934.32 J. However, it depends on the health diseases and age of older adults in the study. The age range of participants in our study is higher than the studies available in the literature.

Among the other approaches that use mobile devices for automation of the Timed-Up and Go text, the most prominent ones are [32,45,49,67]. Similarly, our study also measures the duration of the Timed-Up and Go test and identify the different stages. Unlike them, our study is mainly performed by older adults, uses multiple sensors to monitor the various movements, and measures parameters including power, velocity, acceleration, force, reaction time, and others, to measure the performance of the test more accurately. The main differences and advantages of our study are presented in Table 5.

## 5. Conclusions

The Timed-Up and Go test is an easy test used to measure different types of mobility. This study considered performed the analysis of older adults. This test consists of the individual sitting on the chair, getting up from the chair, walking three meters, reversing the direction of the walking, walking another three meters to back to the chair, and sitting on the chair. 

The automatic measurement of the Timed-Up and Go test with mobile devices is possible, validating the different parts of the test. This work considers the data acquired from the various sensors available in the mobile device, including the accelerometer and magnetometer sensors, where the magnetometer sensors help in the detection of the changes of the direction during the test, where the accelerometer sensors allow the measurement of the acceleration, velocity, force, and power. A Bitalino device with a pressure sensor in the chair is used to detect the mobility’s start. Another Bitalino device was used to acquire the electrocardiography (ECG) and electroencephalography (EEG) for future processing.

This work aimed to analyze the data obtained in different elderly institutions with various conditions. It was verified that data acquisition conditions influenced data acquisition. The different diseases of the individuals also affect the results of the performance of the Timed-Up and Go test. Through the automatic calculation of the features, different values were obtained. Thus, various analyses were carried out by age, institution, and type of disease, which allowed the measurement of exciting results. It was verified that this study allows the possibility to create different patterns of physical states of people. However, several constraints may have influenced the experiment’s results, including the test environment and the reception conditions of the network. The data are somewhat heterogeneous because we are analyzing older adults with different health conditions. The statistical grouping by different age ranges allows us to show the influence that age may have on the test results. The Timed-Up and Go test has been demonstrated to be an accessible and clinically relevant test to assess mobility, balance, and risk of falls in the elderly and other populations with health problems.

With the rise of chronic health conditions, it is fundamental to create accessible, valid, and reliable online instruments that evaluate and record physical health performance, like the Timed-Up and Go test. It is also vital to guarantee that the follow up gives a real evolution of this performance with some health treatments, such as physiotherapy. Future work may recognize different diseases with the values acquired during the experiments, considering the ECG and EEG sensors. The values obtained with the ECG sensor allow for the detection of dysrhythmias, ischemia, driving disorders, ST-segment abnormality, cavity overload, pericarditis, pericardial effusions, ion disorders, and congenital heart diseases. On the other hand, the values obtained with the EEG sensor allows the detection of convulsions, metabolic encephalopathies, structural encephalopathies, degenerative diseases, infections, sleep disorders, and memory changes. 

This pilot study proved to be a great way to help diagnose different types of diseases, whether they involve the individual’s motor capacity, whether cardiac or neurological. In the future, the use of low-cost systems and mobile sensors may help an evolution in medicine for the diagnostics of different diseases in people.

## Figures and Tables

**Table 1:** Distribution of the different diseases involved in the study.

		Number of Occurrences	Related with Mobility
Osteoarticular diseases (Total of 17 individuals)	Arthrosis	4	Yes
	Scoliosis	2	Yes
	Leg amputation	2	Yes
	Bilateral gonarthrosis	2	Yes
	Osteoarthritis	4	Yes
	Lumbar hernias	1	Yes
	Prosthesis in the right humeral	1	Yes
	Osteoporosis	4	Yes
Cardiovascular diseases (Total of 18 individuals)	Arterial hypertension	16	No
	Cardiac arrhythmia	4	No
	Arteriosclerotic coronary disease	1	No
	Heart failure	5	Yes
	Acute myocardial infarction	1	No
	Chronic Venous Insufficiency of the lower limbs	1	No
Lung diseases (Total of four individuals)	Pulmonary fibrosis	1	No
	Chronic obstructive pulmonary disease	2	Yes
	Chronic bronchitis	2	Yes
Neurological and balance disease (Total of six individuals)	Parkinson	3	Yes
	Dementia	1	Yes
	Chronic headaches	1	No
	Sequelae of surgery to brain injury	1	No
Psychiatric illnesses (Total of six individuals)	Post-traumatic stress	1	No
	Depression	5	No
Nephro-urological disease (Total of nine individuals)	Hypocoagulated	1	No
	Anemia	3	No
	Chronic kidney disease	3	No
	Prostate cancer	4	No
Digestive system and abdominal wall disease (Total of three individuals)	Umbilical hernia	2	No
	Inguinal hernia	1	Yes
	Cirrhosis	1	No
	Gastroenteritis	1	No
Metabolic disorder (Total of 10 individuals)	Hyperuricemia	2	No
	Diabetes mellitus Type II	9	No

**Table 2:** Distribution of the different diseases found in the population by its relation to mobility.

Related to Mobility	Not Related to Mobility
- Arthrosis - Scoliosis - Leg amputation - Bilateral gonarthrosis - Osteoarthritis - Lumbar hernias - Prosthesis in the right humeral - Osteoporosis - Heart failure - Chronic obstructive pulmonary disease - Chronic bronchitis - Parkinson - Dementia - Inguinal hernia	- Arterial hypertension - Cardiac arrhythmia - Arteriosclerotic coronary disease - Acute myocardial infarction - Chronic Venous Insufficiency of the lower limbs - Pulmonary fibrosis - Chronic headaches - Sequelae of surgery to brain injury - Post-traumatic stress - Depression - Chronic anemia - Hypocoagulated - Anemia - Chronic kidney disease - Prostate cancer - Umbilical hernia - Cirrhosis - Gastroenteritis - Hyperuricemia - Diabetes mellitus Type II

**Table 3:** Relation between sensors and results obtained.

Sensors	Parameters	Analysis		
		By Age	By Institution	By Diseases
Pressure sensor	Reaction time	-	It is higher in Lar Aldeia de Joanes and Lar Minas (14.860 s), and lower in Lar Nossa Senhora de Fátima (5.948 s)	It is higher in persons with sequelae of surgery to brain injury (16.830 s), and lower in persons with pulmonary fibrosis, acute myocardial infarction, and hypocoagulated (3.477 s)
	Total test time	It is lower in an individual of 60-years-old with scoliosis (21.070 s)	-	It is higher in an individual with a leg amputation and diabetes mellitus Type II (92.950 s).
Magnetometer sensor	Total test time	It is lower in an individual of 60-years-old with scoliosis (19.761 s)	It is lower in Centro Comunitário das Lameiras (28.778 s), and higher in institutions with poor conditions (74.053 s)	It is higher in people with osteoarticular pathology and a prosthesis in the right humeral (66.947 s), and lower in people with arthrosis (24.528 s)
	Turnaround measured by the magnitude of the vector	The time is higher in an individual of 89-years-old with problems related to mobility (51.742 s)	The instant is lower in Lar da Misericórdia (2.591 s)	The instance is higher in people with congestive heart failure (28.886 s), and lower in people with osteoarticular pathology and prosthesis in the right humeral (3.836 s), and the time is higher in people with lumbar hernias and a gastric ulcer (30.643 s)
	Turning around instant measured by the absolute value of the *z*-axis	It is higher in participants with osteoarthritis of 87-years-old (39.649 s).	It is lower in Centro Comunitário das Lameiras (8.433 s), and it is higher in Lar Nossa Senhora de Fátima (39.649 s).	It is lower in people with osteoarticular pathology and a prosthesis in the right humeral (8.704 s), and it is higher in people with osteoarthritis (39.649 s)
Accelerometer sensor	Times	Average of 10.521 s in reaction time, 45.538 s in total test time, 13.272 s in going time, and 21.944 s in return time		
	Turning around	In average, the duration is 0.436 s, and the instant is 23.566 s		
	Acceleration	Average of 9.96 m/s^2^ in going time, and −11.43 m/s^2^ in return time.		
	Velocity	Average of 15.12 m/s in going time, and −5.51 m/s in return time.		
	Force	Average of 713.37 N in going time, and −1886.03 N in return time.		
	Power	Average of 6233.21 J in going time, and −8491.09 J in return time.		

**Table 4:** Relation between the origin and limitations of the study.

Origin	Limitation
Individuals	Different health conditions.
Environment	The experiments were performed in uncontrolled environments.
Technical	The Internet connecting is needed for data synchronization.
	Bluetooth connected reported some failures.
	A large volume of data needs to be processed in the mobile device.
	Data cannot be processed in real-time.
	Sometimes it was not possible to consistently synchronize the timestamps of the acquired data, because Bitalino does not have real timestamps.

**Table 5:** Comparison of the studies in the literature with our study.

Study	Differences Compared to Our Study	Advantages of Our Study
[45]	The study is related to the fall risk assessment, and our research is associated with the analysis of the performance of the Timed-Up and Go test for the creation of patterns by age, disease, and institution.	Our study proved that a relation between diseases related to mobility and the performance of the Timed-Up and Go test exists, allowing the creation of different patterns with the inertial sensors.
[49]	The study identified the different phases of Timed-Up and Go sensors. The authors also calculated the Minimal Detectable Change based on the speed, where we identified the various stages, and measured the force, power, and acceleration of the movement.	The older adults sometimes performed more force and power than the other population. The measurement of these parameters is vital to identify the reliability of the test in the different repetitions.
[32]	The study tracks the different stages of the Timed-Up and Go test, and the angles of the knee and ankle. Our study identified the different phases and made other measurements.	Our study is focused on older adults that commonly have different pathologies, performing different measurements and relationships between diseases.
[67]	The authors implemented machine learning methods for the distribution of the individuals in different groups to cluster the types of diseases.	Our study performed the analysis of the different features extracted with a focus on the diseases related to the movement.

## Appendix A

This section presents Table A1 related to the features extracted in different studies. Also, it presents Table A2 related to the description of the population of the study.

**Table A1:** Studies vs. Features extracted.

Features	Studies	Number of Studies
Duration of the test	[17,32,49,51,52,54,55,58]	8
Maximum	[17,45,56,57,58]	5
Mean	[46,49,54,56,58]	5
Duration of each stage	[17,47,50,51,56]	5
Root Mean Square (RMS)	[45,46,56,58]	4
Standard deviation	[45,46,56,58]	4
Velocity	[32,44]	2
Time of sit-to-stand transition	[44,48]	2
Minimum	[45,57]	2
Energy	[45,46]	2
Entropy	[45,46]	2
Mobility angles	[32,48]	2
Time of stand-to-sit	[53,55]	2
Time of prepare-to-sit	[53,55]	2
Time of sit-down	[53,55]	2
Time of lift-up	[53,55]	2
Maximum change of the trunk angle	[51,55]	2
Maximum angular velocity during the lean forward and lift-up phases	[51,55]	2
Median deviation	[45]	1
Skewness	[45]	1
Interquartile range (IQR)	[45]	1
Kurtosis	[45]	1
Maximum and second maximum frequencies and amplitudes of the Fast Fourier Transform (FFT)	[45]	1
Number of times that the amplitude of the magnitude of the vector of accelerometer signal crosses the mean value	[45]	1
Mean of peak height	[45]	1
Correlation	[46]	1
Pitch	[46]	1
Signal Magnitude Area (SMA)	[46]	1
Signal Vector Magnitude (SVM)	[46]	1
Angular velocity of the mobility of the arm	[50]	1
Time to perform turn-to-sit	[50]	1
Time of lean forward phase	[53]	1
Time of the walking phase	[53]	1
Maximum angular velocities during lean forward and lift-up phases	[53]	1
Maximum change of trunk angle during the lean forward phase	[53]	1
Total number of steps during the walking phase and before the turn	[53]	1
Stride length	[32]	1
Distance traveled	[32]	1
Length of the lean forward period	[55]	1
Number of steps during	[55]	1
Coefficient of variation	[56]	1
Jerk	[58]	1

**Table A2:** Description of the population of the study and test conditions.

Institution	Person ID	Diseases	Diseases Related to Mobility	Age (Years)	Test Conditions
Centro Comunitário das Lameiras	1	Arthrosis	Yes	85	Chair without supports. Spacious place. Floor with the right conditions. Good mobile network coverage. A physical therapist monitored the test.
Centro Comunitário das Lameiras	2	Gastroenteritis	No	92	
Centro Comunitário das Lameiras	3	Arterial hypertension; Arthrosis	Yes	85	
Centro Comunitário das Lameiras	4	Arterial hypertension; Cardiac arrhythmia	No	92	
Centro Comunitário das Lameiras	5	Arterial hypertension; Cardiac arrhythmia; Diabetes mellitus Type II; Scoliosis	Yes	92	
Centro Comunitário das Lameiras	6	Scoliosis	Yes	85	
Centro Comunitário das Lameiras	7	Osteoporosis	Yes	83	
Centro Comunitário das Lameiras	8	Arthrosis	Yes	87	
Others	9	Scoliosis	Yes	60	Excellent quality of mobile network coverage. Tight space in the kitchen. Chair with supports.
Others	10	Right leg amputation; Diabetes mellitus Type II	Yes	77	
Lar Aldeia de Joanes	11	N/D	-	N/D	Weak mobile network coverage. Test site with the right physical conditions. The test was carried out in a place with other older adults. Chair with supports.
Lar Minas	12	Arterial hypertension	No	88	Mobile network coverage does not exist. Test site with Good physical condition of the test site. The test was carried out in a living room with other older adults. Chair with supports.
Lar Minas	13	Arterial hypertension; Cardiac arrhythmia; Arteriosclerotic coronary disease; Heart failure	No	84	
Lar Minas	14	N/D	-	65	
Lar da Misericórdia	15	N/D	-	91	The basement of a building with little mobile network coverage. Chair with supports. Flat ground with a slight slope.
Lar da Misericórdia	16	N/D	-	84	
Lar da Misericórdia	17	Hernioplasty in 2010; Sarcoidosis	No	87	
Lar da Misericórdia	18	Chronic obstructive pulmonary disease; Chronic bronchitis; Osteoarthritis	Yes	73	
Lar da Misericórdia	19	Cirrhosis; Anemia; Chronic kidney disease; Umbilical hernia; Inguinal hernia	Yes	79	
Lar da Misericórdia	20	Right leg amputation; Umbilical hernia; Arterial hypertension	Yes	88	
Lar da Misericórdia	21	Prostate Cancer; Parkinson’s disease; Post-traumatic stress	Yes	76	
Lar da Misericórdia	22	Arterial hypertension; Diabetes mellitus Type II	No	86	
Lar da Misericórdia	23	Prostate Cancer; Osteoporosis; Chronic Venous Insufficiency of the lower limbs; Chronic bronchitis	Yes	92	
Lar da Misericórdia	24	Diabetes mellitus Type II; Arterial hypertension; Depression; Sequelae of surgery to brain injury	No	83	
Lar da Misericórdia	25	Diabetes mellitus Type II; Vertigo syndrome; Chronic headaches; Osteoarthritis; Prosthesis in the right humeral; Osteoporosis; Arterial hypertension	Yes	81	
Lar da Misericórdia	26	Arterial hypertension; Anemia	No	91	
Lar da Misericórdia	27	Osteoarthritis; Depression; Heart failure; Arterial hypertension; Osteoporosis	Yes	89	
Lar da Misericórdia	28	N/D	-	N/D	
Lar da Nossa Senhora de Fátima	29	Diabetes mellitus Type II;	No	86	The test location was narrow. The mobile network coverage was of good quality. The floor and width of the test site were very tight. The chair had no supports.
Lar da nossa senhora de Fátima	30	Dementia of vascular etiology; Prostate Cancer; Arterial hypertension; Vertigo syndrome	Yes	N/D	
Lar da nossa senhora de Fátima	31	Depression; Osteoporosis	Yes	83	
Lar da Nossa Senhora de Fátima	32	Diabetes mellitus Type II; Osteoarthritis	Yes	87	
Lar da Nossa Senhora de Fátima	33	Diabetes mellitus Type II; Arterial hypertension; Heart failure; Hyperuricemia; Depression; Bilateral gonarthrosis	Yes	N/D	
Lar da nossa senhora de Fátima	34	Prostate cancer	No	88	
Lar da Nossa Senhora de Fátima	35	Heart failure; Chronic obstructive pulmonary disease; Bilateral gonarthrosis	Yes	97	
Lar da nossa senhora de Fátima	36	Diabetes mellitus Type II; Arterial hypertension	No	71	
Lar da nossa senhora de Fátima	37	Arterial hypertension	No	74	
Lar da Nossa Senhora de Fátima	38	Osteoarthritis; Lumbar hernias; Depression; Gastric ulcer	Yes	82	
Lar da Nossa Senhora de Fátima	39	Heart failure; Arterial hypertension; Pulmonary fibrosis; Hyperuricemia; Anemia; Chronic kidney disease; Cardiac arrhythmia; Acute myocardial infarction; Hypocoagulated	Yes	N/D	
Lar da nossa senhora de Fátima	40	Chronic kidney disease	No	90	

N/D: The values were not reported by the older adults.
